# Gene expression profile of chronic oral graft-versus-host disease

**DOI:** 10.1371/journal.pone.0267325

**Published:** 2022-04-29

**Authors:** Giselle Rocha Pinto, Viviane Almeida Sarmento, Paulo Cirino de Carvalho-Filho, Vitor Antonio Fortuna, Ryan dos Santos Costa, Rogério Reis Conceição, Soraya Castro Trindade

**Affiliations:** 1 Department of Dentistry, Federal University of Bahia (UFBA), Salvador, Bahia, Brazil; 2 Department of Dentistry, School of Medicine and Public Health, Salvador, Bahia, Brazil; 3 Health Science Institute, Federal University of Bahia (UFBA), Salvador, Bahia, Brazil; 4 Department of Dentistry, Feira de Santana State University (UEFS), Feira de Santana, Bahia, Brazil; Indiana University School of Medicine, UNITED STATES

## Abstract

Among the complications observed after allogeneic hematopoietic stem cell transplantation, graft-versus-host disease (GVHD) is the primary cause of post-transplant mortality. The oral cavity is the second most affected organ target in chronic GVHD. Tissue damage results from the upregulation of inflammatory mediators, which play a critical role in the immunopathogenesis of the disease. This case series observational study aims to evaluate the participation of cytokines, chemokines, transcription factors, and heat shock proteins in the pathogenesis of oral GVHD (oGVHD), describing the mRNA expression of 28 genes selected. Peripheral blood mononuclear cells were isolated from six participants with oGVHD and two without GVHD, and relative expression of transcripts with established roles as inflammatory mediators was determined in triplicate using the human RT^2^ Profiler™ PCR Array. The gene expression levels in the group with oGVHD were mainly up-regulated compared to those without GVHD. PBMC from oGVDH expressed consistently higher *IFN-γ*, *TNF*, *IL-1β*, *CCL2*, *HSP60 (HSPD1)* and *HSP90 (HSP90B1)*. These results can provide a basis for developing new molecular diagnostics and targets therapies for the clinical management of oGVHD.

## Introduction

Allogeneic hematopoietic stem cell transplantation (HSCT) has evolved as effective cellular immunotherapy to treat many hematologic and primary immunodeficiency disorders [1]. Chronic graft-versus-host disease (cGVHD), an auto and alloimmune systemic disorder, is a severe and potentially fatal complication post-HSCT. Although the precise mechanism is unclear, chronic GVHD is generally recognized as a T cell–mediated disorder. It involves mixed infiltrating inflammatory cells and the production of allo- and auto-antibodies by aberrant B cells that exacerbate direct or indirect inflammatory responses [2, 3]. A defective immune tolerance in the hosts after allo-HSCT might also favor progress and the occurrence of chronic GVHD [4].

The oral cavity is among the most commonly affected region in chronic GVHD, second only to the skin [5, 6]. The clinical manifestation of oGVHD comprises the epithelium, connective tissue and minor salivary glands. The oral condition is usually mild and characterized by lichenoid lesions and hyperkeratotic plaques, though moderate to severe erosive and ulcerated lesions, mucosal atrophy, salivary gland dysfunction, mucoceles, reduced mouth opening, sensitivity to oral feeding, and dryness of mouth may also be seen [5].

The complex and multifactorial nature of oral GVHD and limited access to biological specimens make the study of the mechanisms involved in the immunopathogenesis of human oral GVHD particularly challenging. Both the T cells and B lymphocytes are implicated in the loss of general immune tolerance, although few studies focused on oral GVHD. A study by Imanguli et al., 2009 has shown that oral cGVHD resulted from the activation of the type-I IFN axis with local immigration, proliferation, and differentiation of T effectors cells [7]. Recently, the loss of dental microbiota was associated with the risk and intensity of acute GVHD but not with other allo-HSCT outcomes, such as oral chronic GVHD [8]. In contrast, Yong et al., 2021 suggest that serum cytokines rather than cytokine-secreting cells were involved in driving and maintaining oral GVHD [9].

Therefore, this study investigated the participation of cytokines, chemokines, transcription factors and heat shock proteins in the pathogenesis of oGVHD, describing the expression of 28 genes in the peripheral blood mononuclear cells (PBMC) and their relationships with the oral manifestations in cGVHD patients.

## Material and methods

This case series observational study was developed according to the Declaration of Helsinki. The institutional review board of the Professor Edgard Santos University Hospital approved this study (Federal University of Bahia, approval n° 3.573.494). All participants gave written informed consent.

### Study participants

Eight participants (five men and three women) were enrolled after allogeneic hematopoietic stem cell transplantation in this study and followed in the hematology outpatient facility in 2018. Exclusion criteria were (1) age below 18 years, (2) pregnancy, and (3) people with other autoimmune diseases or infections.

Chronic GVHD diagnosis followed the National Institutes of Health Consensus Criteria and histopathology confirmation [10]. Oral examination of cGVHD patients and biopsy collection occurred during the Dental Clinic facility monitoring. Six participants diagnosed with oral cGVHD and two patients without GVHD (reference) were investigated. The differences between demographic and clinical data groups are shown in the S1 Table.

At the time of blood collection, two patients with oGVHD had oral lesions (active GVHD), and four patients had completely healed mucosa (inactive GVHD).

### Blood collection

Venous blood (20 mL) was collected in heparinized vacutainers and submitted to density gradient centrifugation to isolate PBMC, using the protocol according to the manufacturer’s instructions (Histopaque®-1077, Sigma-Aldrich Co., St. Louis, MO, USA).

### Gene expression analysis by quantitative reverse transcriptase polymerase chain reaction

Total RNA was isolated using the *NucleoSpin RNA* kit (Macherey-Nagel GmbH & Co. KG, Düren, Germany). The cDNA was synthesized using 1 μg of the total RNA with the *QuantiTect Reverse Transcription* kit (Qiagen, Valencia, California, USA). The resulting cDNAs were amplified using the Custom Human RT^2^ ProfilerTM PCR Array CAPH12794 (SABiosciences, Frederick, MD, EUA) in 384-well plates using the thermocycler Applied Biosystems QuantStudio™ 12K Flex Real-Time PCR System 384-well block (Applied Biosystems), according to manufacturer’s instruction. Twenty-eight different genes were simultaneously analysed, and a panel of three housekeeping genes normalized the PCR Array data (S2 Table).

### *In silico* analysis

In addition, *in silico* analysis employing the Search Tool for the Retrieval of Interacting Genes/Proteins (STRING) database was conducted to map out the interaction of proteins whose genes were up-regulated in the patients with oral GVHD in comparison to those without GVHD.

### Data analysis

The fold change and relative expression levels to reference were calculated according to the previously described ΔΔCt method [11], using an online data analysis RT^2^ Profiler™ PCR Array Data Analysis software—SABiosciences (http://dataanalysis.qiagen.com/pcr/arrayanalysis.php). A heat map was constructed to provide a graphical representation of the gene expression profile of the molecules studied. The group without GVHD was used as a reference.

## Results

The mean age of oral GVHD patients was 48 years (three males and three females), while the reference group was 33 years (2 males). Clinically, most patients with oral GVHD presented mucosal atrophy and erosive thinning due to previous lesions (n = 4) (inactive GVHD). Active lesions, such as ulcerations and linear gingival erythema, were observed in 2 patients at the biopsy collection (active GVHD).

**Fig 1:**
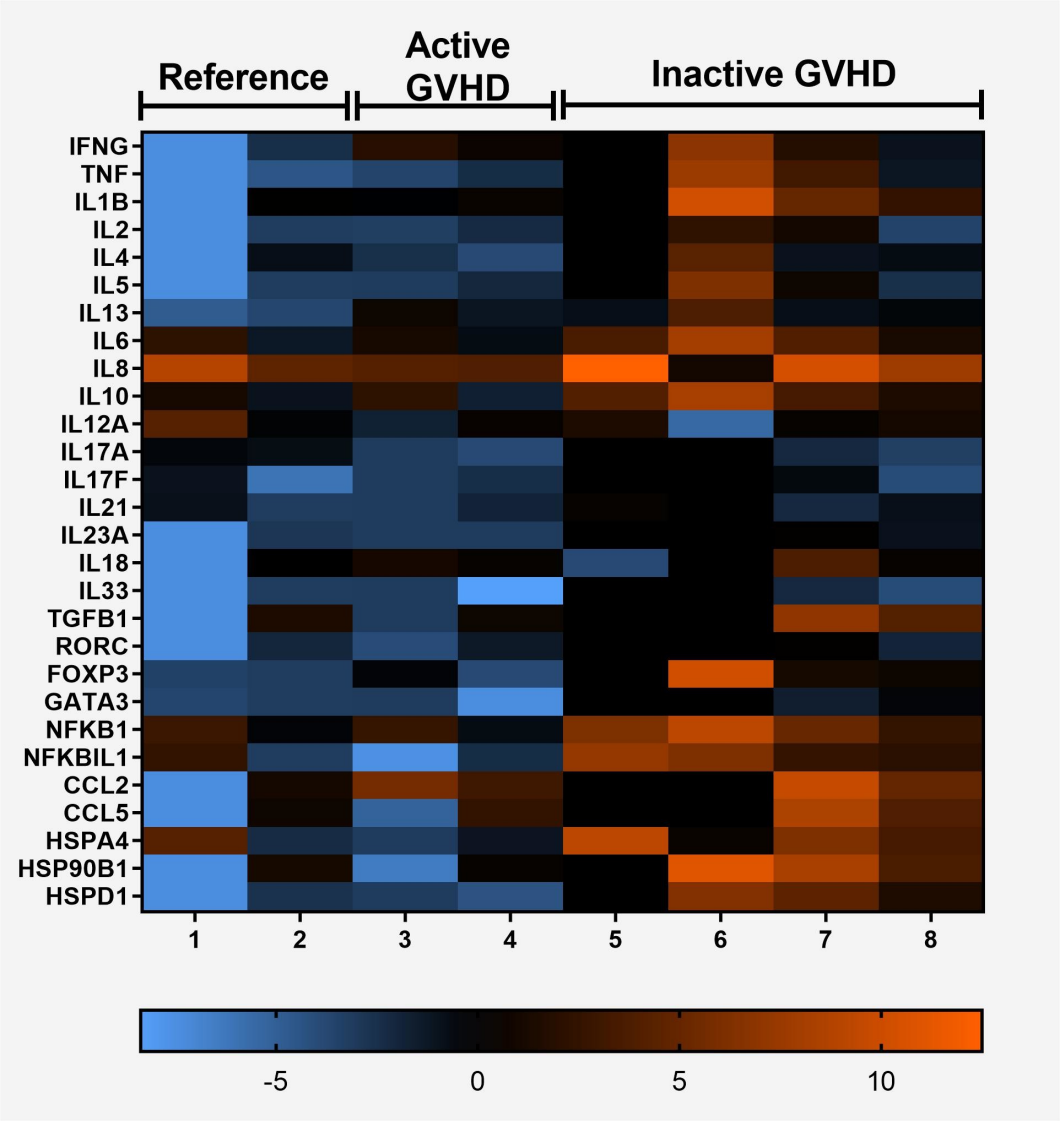
Heat map depicting the expression levels of 28 selected genes. High (orange) and low (blue) expression levels according to the scale at the bottom of the heat map. Up-regulated and down-regulated genes in oral GVHD with an inactive lesion (n = 4) compared to the oral GVHD with an active lesion (n = 2) and with the reference group (n = 2). GAPDH was selected for the normalization of the quantitative RT-PCR data. Gene names are on the right side of the heat map.

To explore whether gene expression could define a predictive molecular signature for the oral GVHD patients, we isolated total mRNA from PBMC samples and monitored the expression of immune-response and inflammation-specific genes with a qPCR-microarray system. Consistent with the notion that oral GVHD strongly correlates with chronic GVHD, we observed that most genes analyzed were up-regulated (19 out of 28) and only one gene was down-regulated in oral GVHD patients (Fig 1). However, a substantial proportion of genes (9 out of 28) were expressed in oral GVHD patients with active ulcerated lesions in different magnitudes than inactive oral lesions.

### Relative gene expression profile in oral GVHD

**Fig 2:**
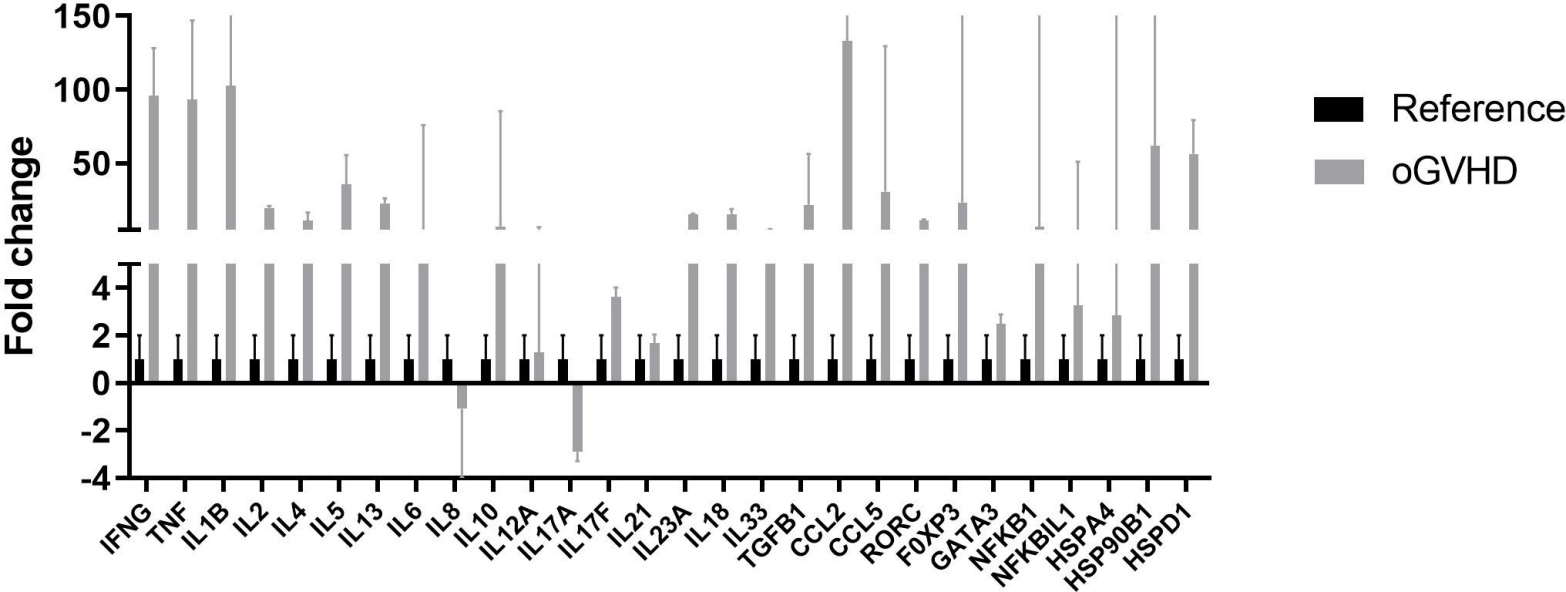
Gene expression in the oGVHD and reference groups. Relative expression of cytokines, chemokines, HSP and transcription factors in PBMC from the oral GVHD group (n = 6) relative to the reference group (without GVHD, n = 2). Gene expression was normalized to the housekeeping gene (GAPDH), and the mean (± SD from 2 replicates) expression relative to the reference group was plotted and presented on a linear scale.

**Fig 3:**
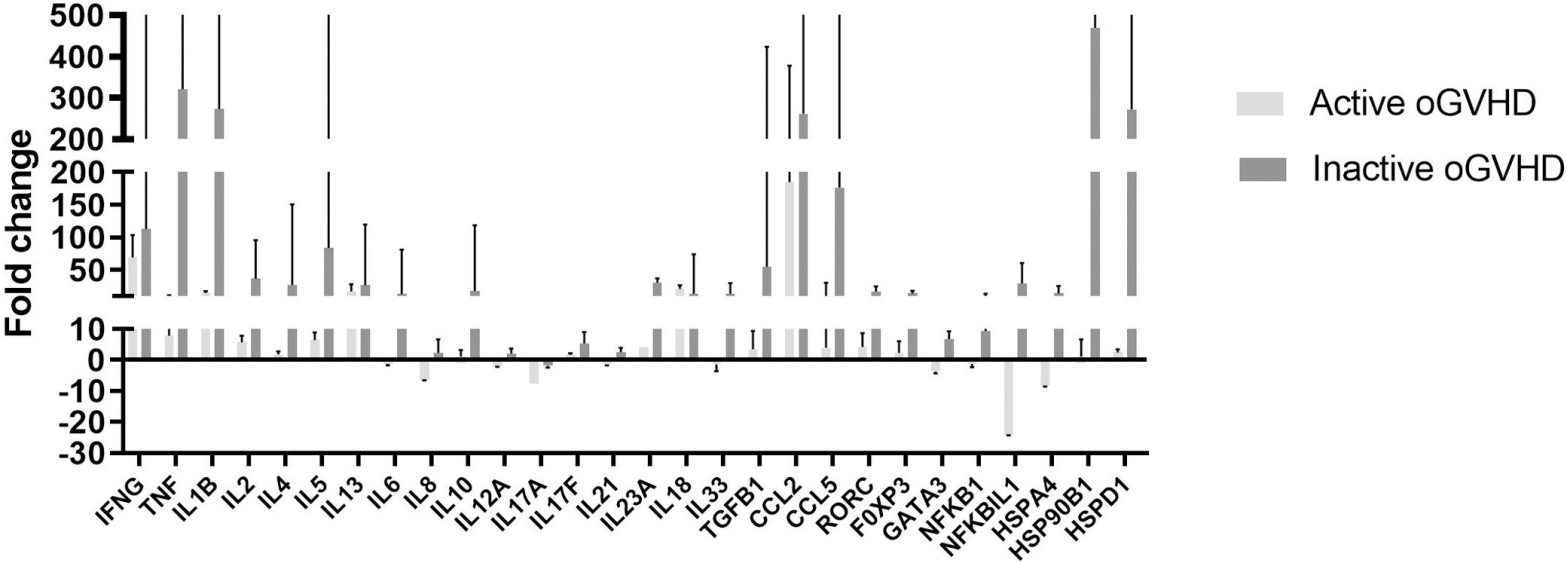
Gene expression in the active and inactive GVHD subsets. Relative expression of cytokines, chemokines, HSP and transcription factors in PBMC from the active oral GVHD subset (n = 2) relative to the inactive GVHD subset (n = 4). Gene expression was normalized to a housekeeping gene (GAPDH), and the mean (± SD from 2 replicates) expression was plotted and presented on a linear scale.

The profile of cytokines, chemokines, transcription factors, and heat shock proteins was mainly up-regulated in the oral GVHD group (Fig 2) and the subset of patients with inactive oral lesions compared to the active lesion subset (Fig 3). The changes in the relative gene expression of *IFN-γ*, *TNF*, *IL-1β* and *CCL2* were consistently more remarkable (100-fold on average), while the *IL-17A* expression level was down-regulated in both active and inactive oral GVHD.

*HSPD1* (60 kDa) and *HSP90B1* (90 kDa) were also highly expressed (~ 60-fold higher) in the oral GVHD group (Fig 2) and the subset of patients with inactive oral lesions in comparison to the active lesion subset (Fig 3).

Transcription factors with higher expression in the oral GVHD group included *FOXP3* (25-fold) (Fig 2), while in the oral GVHD subset with inactive GVHD also included RORC (18-fold) and GATA3 (8-fold) (Fig 3).

### Gene interaction network

**Fig 4:**
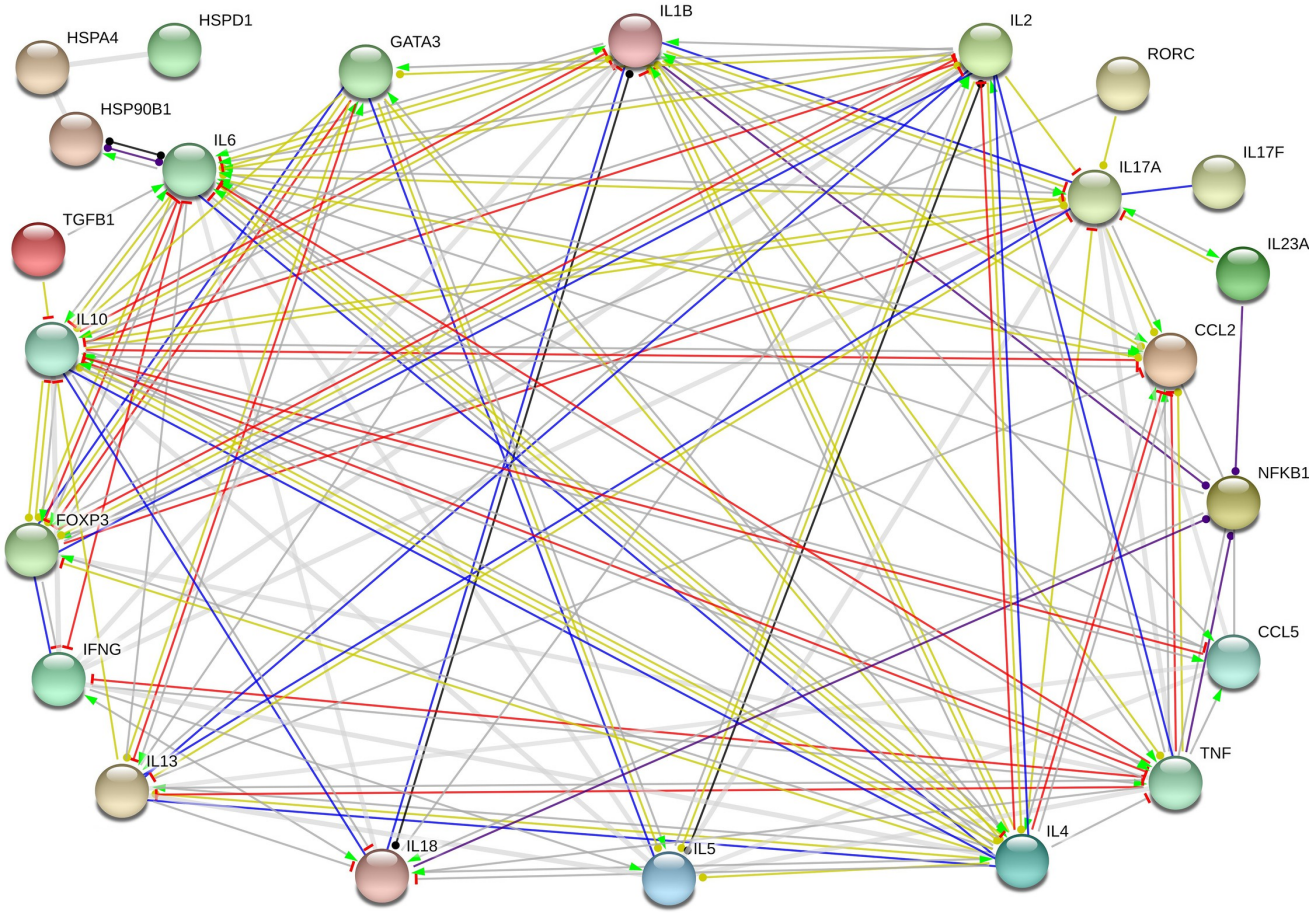
Gene interaction network. *In silico* protein-protein interaction network of up-regulated genes in patients with oGVHD and patients without GVHD. The nodes between genes represent the protein-protein interaction pairs from the STRING (Search Tool for the Retrieval of Interacting Genes/Proteins) database. Edges represent protein-protein associations. Actions types: green–activation, blue–binding, black–reaction, red–inhibition, purple–catalysis, yellow–transcriptional regulation. Source: STRING interaction network.

We explored the functional network of genes and the predicted way they interact as shown in Fig 4. The cytokines connected in the interactome present well-known interactions strongly associated with the actions of inhibition, connection and transcriptional regulation (red, blue and yellow lines, respectively). A weaker interaction predicted between HSPA4, HSPD1 and HSP90B1 proteins occur through reaction and catalysis in an undefined manner and positive activation with IL-6.

STRING protein association network predicted intense activation between the transcription factors GATA3 and FOXP3 and Th2 response cytokines. There was also an inhibitory relationship between FOXP3 and IL-17A or IL-6, and a functional catalytic activation relationship between NFκB1 and IL-23, IL-1β, IL-8, and TNF. There was no predicted interaction involving NFκBIL1.

## Discussion

In the present study, of all 28 genes examined, the transcriptional level of 20 genes showed more than a ten-fold difference between presence and absence of oral GVHD, agreeing with the findings of Yong et al., 2021, that highlight the importance of serum cytokines for the development of oGVHD [9]. *TNF*, *IL-1β*, *IFN-γ* and *CCL2* show the highest expression level in the oGVHD group. Because of the paucity of available tissue, little information has previously been available regarding the gene expression profiles of oGVHD. To our knowledge, this is the first time the up-regulation of these genes has been associated with oGVHD.

The increase in TNF and IFN-γ suggests T polarization toward the Th1 phenotype, complementing prior findings on oGVHD [7]. Previously, TNF was often associated with the presence or increased risk of developing GVHD [12–14] Similarly, the increased expression of IFN-γ was related to cGVHD [13, 14] and oGVHD [15]. However, there is also evidence that IFN-γ can inhibit GVHD and facilitate graft-versus-leukemia effects [16]. Depending on the target organ, IFN-γ can be a protector or inflammatory factor.

IL-1β, which plays a crucial role in immune responses and induces the development of Th17 lymphocytes, was also previously associated with cGVHD [17, 18].

Regarding CCL2, its plasma concentration seems to increase with a rise in alloreactive T cells in pulmonary and hepatic GVHD [19, 20], but this effect was not observed in the intestine [21], showing once again that affected tissues have their peculiarities. It is important to note that oGVHD is primarily driven by T lymphocytes, with the participation of macrophages [22]. The association between macrophage migration and adhesion and oGVHD is mediated, in part, by CCL2/ CCR2 [23]. CCR2+ macrophages are generally M2 or activated type [24] and can be associated with the pro-fibrotic characteristics of oGVHD.

The interactome analysis described in this study demonstrated that these four proteins (TNF, IL-1β, IFN-γ and CCL2) contribute to shared functions through activation, and post-transcriptional regulation, indicating that they are acting in an interconnected manner in the oGVHD pathogenesis.

*CCL5* and *IL-5* also stand out due to their gene expression in individuals with oGVHD. T lymphocytes are recruited at the lesion site and produce large quantities of these molecules, confirming that they have an essencial role in maintaining and prolonging the immune response, contextualizing its participation in oGVHD in this study [15, 24].

Curiously, in the present study, there was an up-regulation of *IL-17F* in oGVHD but a down-regulation of *IL-17A*. Although both have previously been associated with cGVHD [25], it has been shown that IL-17A is capable of suppressing the production and secretion of IL-17F [26] and that IL-17A-producing CD4 T (Th17) cells induce GVHD, whereas the IL-17A cytokine is protective in GVHD after allogeneic bone marrow transplantation [27]. Even though the literature discusses the participation of IL-17 in GVHD, no previous studies evaluated oGVHD specifically. The up-regulation in the relative gene expression of the *RORγt* transcription factor reinforces the participation of the Th17 profile.

The relative gene expression of *HSP60 (HSPD1)* and *HSP90 (HSP90B1)* also stood out in the present study, despite HSP90B1 being its only link to the interaction network. HSP90 has an anti-apoptotic function and is responsible for stabilizing various signaling pathways crucial for the activation of T cells. As such, the selective inhibition of HSP90 in alloreactive T cells has emerged to prevent and treat GVHD [28, 29]. However, more studies concerning oGVHD are necessary.

*FOXP3* showed the highest up-regulation among the transcription factors studied in individuals with oGVHD. This finding diverges from studies on cGVHD, in which T-regulatory lymphocytes (Tregs), which express *FOXP3* constitutionally, are considered fundamental for maintaining tolerance to cGVHD and its suppression [30–32]. The increased gene expression found in this study may not reflect an increased Treg count in patients with oGVHD. Treg lymphocytes have higher proliferation levels than conventional T cells in response to lymphopenia after HSCT, but they are also more susceptible to apoptosis mediated by FAS, resulting in a relative deficiency of Tregs in extensive cGVHD [33].

This study was innovative because it employed a micro-array technique that allowed for a broader investigation of gene transcription and an *in silico* analysis of the interaction between proteins that might be produced in not broadly studied disease, oGVHD. However, it is necessary to be cautious when interpreting the results due to their descriptive nature, given that the number of participants was limiting. Moreover, the experimental groups slightly differ in the mean age, contributing to differential inflammatory molecule levels.

Nonetheless, it was possible to detect some variations in the profile of the two individuals with active oGVDH compared to the other participants in the oGVHD group, corroborating the methodological approach. Also, it should be emphasized that the clinical diagnosis of the lesions in these two individuals was prompt, confirmed histopathological results and that the individuals received no treatment for the disease, all which distinguishes them clinically from the rest of the participants. In addition, the follow-up of the active oGVHD patients after the study showed a significant worsening of their clinical condition, raising the hypothesis that the expression of the genes studied were also increasing over time. Prospective studies should be conducted to confirm this hypothesis.

In patients with inactive oGVHD, a dysregulated immune response was observed even after healing the oral lesions. Both pro-inflammatory and regulatory genes were equally overexpressed in these individuals. Could this be a new pattern of homeostasis in inactive GVHD individuals? It is interesting to reassess these individuals regarding the use of medications, lifestyle, and epigenetic factors that could explore this hypothesis.

## Conclusion

Based on these considerations, we can reason that the the immunopathogenesis of oGVHD probably involves the expression of *IFN-γ*, *TNF* and *IL-1β*. Since these genes were up-regulated, the chemokine *CCL2*, the *HSP 60* and *90*, and transcription factor *FOXP3* expression may also be implicated. This study presents what would seem to be a promising line of investigation that can help in better understanding the pathogenesis of oGVHD and ultimately improve strategies for the prevention and treatment of this disease.

## Supporting information

S1 Table Demographic and clinical characteristics of transplantation patients. (TIF)

S2 Table Genes tested. (TIF)
