# Supplementary figures and images for: Gene expression profile of chronic oral graft-versus-host disease
Source: PLoS One. 2022 Apr 29;17(4):e0267325. doi: 10.1371/journal.pone.0267325 (PMC9053775; doi:10.1371/journal.pone.0267325)

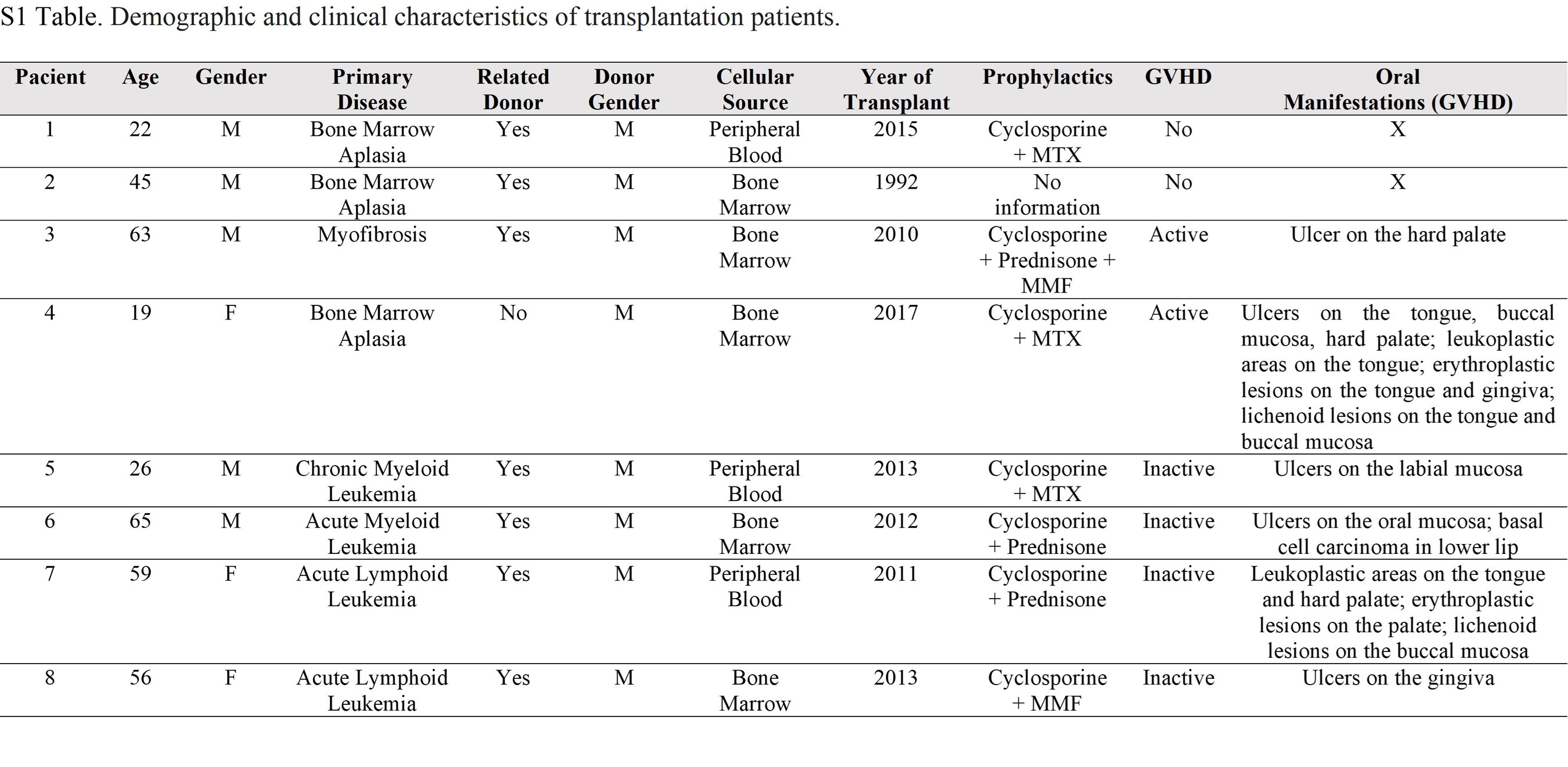

Supplement: S1 Table — (TIF) [file pone.0267325.s001.tif]

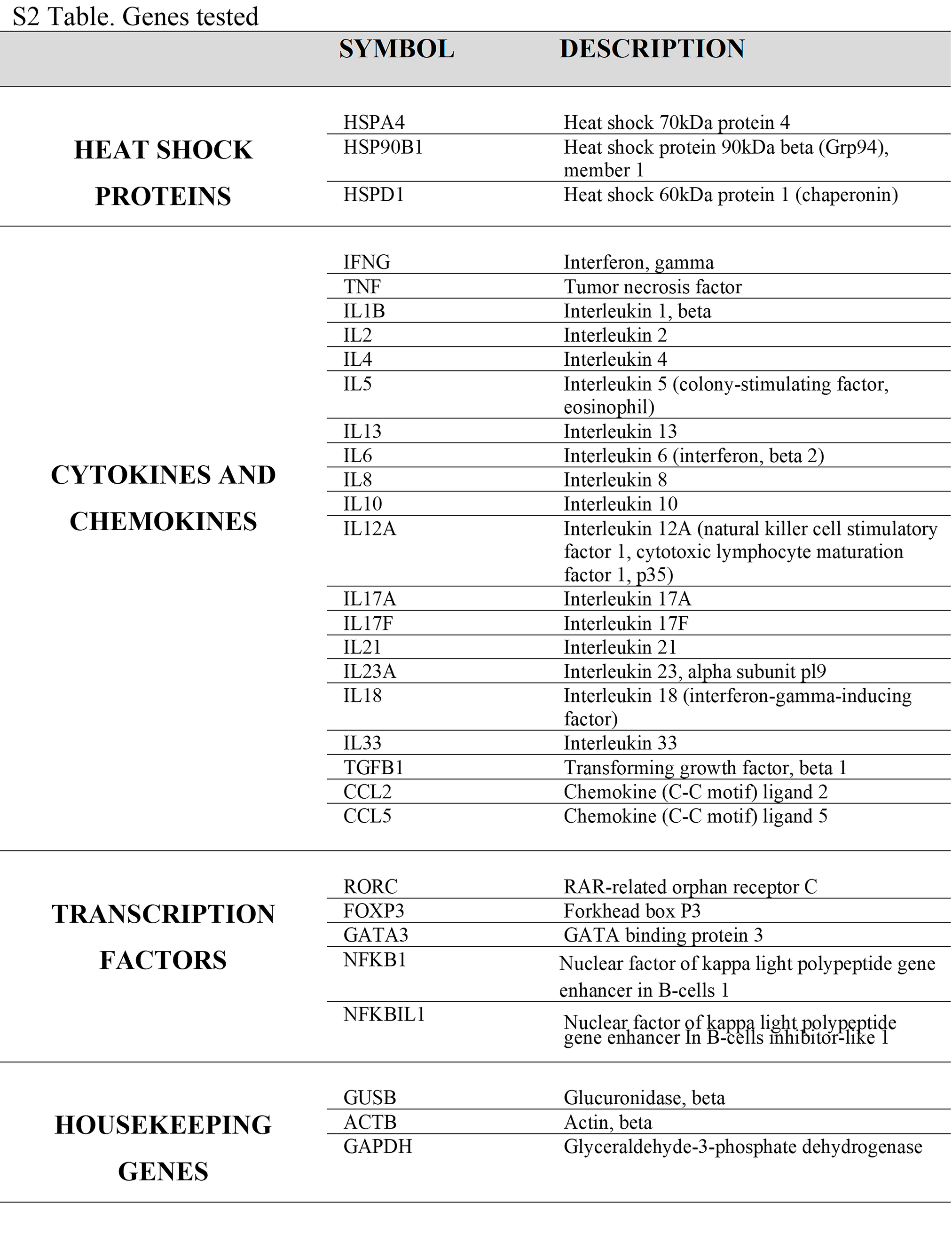

Supplement: S2 Table — (TIF) [file pone.0267325.s002.tif]
